# The mode of school transportation in pre-pubertal children does not influence the accrual of bone mineral or the gain in bone size - two year prospective data from the paediatric osteoporosis preventive (POP) study

**DOI:** 10.1186/1471-2474-11-25

**Published:** 2010-02-03

**Authors:** Bjarne Löfgren, Susanna Stenevi-Lundgren, Magnus Dencker, Magnus K Karlsson

**Affiliations:** 1 Clinical and Molecular Osteoporosis Research Unit, Department of Clinical Sciences, Lund University, Department of Orthopaedics Malmö University Hospital, SE- 20502 Malmö, Sweden; 2 Malmö Clinical Physiology and Nuclear Medicine Unit, Department of Clinical Sciences Lund University, Malmö University Hospital, SE- 20502 Malmö, Sweden

## Abstract

**Background:**

Walking and cycling to school are one source of regular physical activity. The aim of this two years observational study in pre-pubertal children was to evaluate if walking and cycling to school was associated with higher total amount of physical activity and larger gain in bone mineral content (BMC) and bone width than when going by car or bus.

**Methods:**

133 boys and 99 girls aged 7-9 years were recruited to the Malmö Prospective Paediatric Osteoporosis Prevention (POP) study. BMC (g) was measured by dual X-ray absorptiometry (DXA) in total body, lumbar spine (L2-L4) and femoral neck (FN) at baseline and after 24 months. Bone width was measured in L2-L4 and FN. Skeletal changes in the 57 boys and 48 girls who consistently walked or cycled to school were compared with the 24 boys and 17 girls who consistently went by bus or car. All children remained in Tanner stage I. Level of everyday physical activity was estimated by accelerometers worn for four consecutive days and questionnaires. Comparisons were made by independent student's t-tests between means and Fisher's exact tests. Analysis of covariance (ANCOVA) was used to adjust for group differences in age at baseline, duration of organized physical activity, annual changes in length and BMC or bone width if there were differences in these traits at baseline.

**Results:**

After the adjustments, there were no differences in the annual changes in BMC or bone width when comparing girls or boys who walked or cycled to school with those who went by car or bus. Furthermore, there were no differences in the levels of everyday physical activity objectively measured by accelerometers and all children reached above the by the United Kingdom Expert Consensus Group recommended level of 60 minutes moderate to vigorous physical activity per day.

**Conclusion:**

A physical active transportation to school for two years is in pre-pubertal children not associated with a higher accrual of BMC or bone width than a passive mode of transportation, possibly due to the fact that the everyday physical activity in these pre-pubertal children, independent of the mode of school transportation, was high.

## Background

The lifetime risk of sustaining an osteoporotic fracture is very high and lies within the range of 40-50% in women and 13-22% for men [1]. Reports have also inferred that osteoporotic fractures account for 0.83% of the global burden of non-communicable disease and are a significant cause of morbidity and mortality, particularly in the developed countries [2]. This bone fragility may have its foundation in growth [3]. Peak bone mass (PBM) has been suggested as the single most important factor in the development of osteoporosis and a 10% increase in PBM could delay osteoporosis by 13 years [4]. Among the key factors, physical activity has been described as a strategy to optimize skeletal development, as reports have inferred both highly intense [5,6] and moderately intense training [7-9] to increase the accrual of bone mineral. Trials also suggest that the skeletal benefits of exercise can be attained at a population level [10,11]. The school has then been regarded as one arena to launch such programs, as it is one of the few places where all children can be targeted [10,11].

Transportation mode to school has been described as another such possibility. Cross-sectional studies support this when reporting that walking and cycling to school are associated with a higher level of physical activity compared to traveling by vehicle [12,13]. However, to our knowledge no prospective studies have specifically evaluated the hypothesis. The aim of this population based prospective observational study in pre-pubertal children was therefore to evaluate whether walking and cycling to school was associated with higher total amount of physical activity and larger gain in bone mineral content (BMC) and bone width than when going to school by bus or car. We hypothesized that walking and cycling to school is associated with the benefits described above compared to if going by car or bus.

## Methods

We launched in Malmö, Sweden a prospective, controlled exercise intervention study, called The Malmö Paediatric Osteoporosis Prevention (POP) study, which was designed to annually assess musculo-skeletal development in 133 boys and 99 girls from age 7-9 years and onwards. This study has previously extensively been described [10,11]. These reports have shown no differences in age, height, weight or body mass index (BMI) when comparing participants and non-participants in the population based invited cohort of children [10,11], indicating that our results could be generalized. The intervention group was at baseline subjected to 40 minutes of daily physical training within the school curriculum whereas the controls continued with the Swedish mean of 60 min of physical education per week [10,11,14,15]. Baseline measurements were performed at commencement of school and just after the intervention started and follow-up in the same months two years later. A questionnaire evaluated lifestyle factors such as school transportation, duration of organized physical activity, nutritional habits, menarcheal age, medication, fractures and diseases during the study period.

The children included in the POP cohort started in grade 1 or 2 within four different schools in the same region of the city [10,11]. All children in the study population were then pooled and divided into two groups: (1) children who walked or cycled to school and (2) children who travel to school by bus or car. All children, except one boy adopted from Colombia, were Caucasians, without any medication or disease known to influence bone metabolism. All children remained in Tanner stage 1 [16] throughout the study, assessed by our research nurse. Six girls and 5 boys did not answer questions as regard mode of school transportation and 47 boys and 28 girls showed now consistent pattern in their mode of school transportation, leaving 57 boys (24 in the intervention school and 33 in the control schools) and 48 girls (22 in the intervention school and 26 in the control schools) who consistently walked or cycled to school and 24 boys (18 in the intervention school and 6 in the control schools) and 17 girls (11 in the intervention school and 6 in the control schools) who consistently went by bus or car. The mode of school transportation was self selected by the children and their parents. This could be done as public school-transportation is provided by the authorities free of charge without compulsiveness.

Bone mineral content (BMC, g) were measured by dual-energy X-ray absorptiometry (DXA, DPX-L version 1.3z, Lunar^®^, Madison, WI) of the total body, at the lumbar spine (L2-L4 vertebrae and third lumbar vertebra (L3) and at the hip (femoral neck [FN], Ward's region and the trochanter). The width of the L3 vertebra was estimated from the antero-posterior spine scan as the distance from one edge of the L3 vertebra to the other edge. The FN width was calculated from the hip scan as the FN area divided by the scan length of the measured FN area. Total body lean mass and fat mass were estimated from the total body DXA scans. The coefficient of variation (CV) was 1.4-5.2% for BMC, 2.2% for L3 width, 1.5% for FN width, 3.7% for total body fat mass and 1.5% for total body lean mass estimated through duplicate measurements in 13 healthy children aged 7-15 years. All measurements were performed by the research technicians in our laboratory and one of them analyzed all scans. Calibration of the machine was done daily with the standard Lunar^® ^Phantom. Body height was measured to the nearest 0.5 centimetres by a wall-tapered height meter and body weight to the nearest 0.1 kilogram by an electric scale (Avery Berkel model HL 120^®^, Avery Weigh-Tronix Inc., Fairmont, NM, USA).

Accelerometers were used to evaluate the levels of physical activity. The methodology of physical activity measurement has previously been presented in detail [17]. The MTI (Manufacturing Technology Incorporated, Fort Walton Beach, FL, USA) accelerometer, model 7164 was used for four consecutive days. Younger school children exhibits less day-today variability in physical activity than older children, a 4-5 days observation period with accelerometers has been shown to give reliable estimates of usual physical activity behaviour [18].

All accelerometers were calibrated against a standardized vertical movement to minimize inter-instrumental variation. Accelerometer data was averaged over a period called an epoch. In this study a recording epoch of ten seconds was selected. All accelerometer data was analysed using SAS-based software (SAS Institute Inc, Cary, NC, USA), witch automatically deletes missing data defined as continuous sequences of 10 minutes (60 consecutive epochs) or more with zero counts. Such sequences of zeros lasting longer than ten minutes were assumed to be caused by the accelerometer not being worn. Moderate to vigorous physical activity (MVPA) was defined as time spent performing above 3 metabolic equivalents (METs), and time spent performing above 6 METs was considered to reflect vigorous physical activity (VPA). Mean activity was calculated as the total accelerometer counts per valid monitoring minute (counts/min). Age and weight specific cut-off points exist for accelerometer counts representing activity of varying intensities. Cut-off points used for all children were >167 counts/epoch for moderate to vigorous activity and >583 counts/epoch for vigorous activity [19,20]. According to the recommendations made by the United Kingdom Expert Consensus Group, children should accumulate at least 60 minutes of moderate to vigorous physical activity each day [21].

Statistica^® ^version 6.1 (StatWin^®^) was used for statistical calculations and independent Student's *t*-test between means and Fishers exact test were used for comparisons between the groups. Analysis of covariance (ANCOVA) was used to adjust for group differences in age at baseline, duration of organized physical activity, annual changes in length and BMC or bone width if there were differences at baseline in these traits. A *p*-value of < 0.05 was considered as a statistically significant difference. Post-hoc power analysis revealed that an annual difference in gain in BMC L2-L4 of 0.6 g/yrs for girls and 0.5 g/yrs for boys and a gain of 0.3 g/yrs in BMC in FN for girls and 0.2 g/yrs for boys, and in bone width in L3 of 0.6 mm/yrs for girls and of 0.5 mm/yrs for boys and in FN of 0.8 mm/yrs for girls and 0.5 mm/yrs for boys would render statistical significance.

Due to technical difficulties when scanning small children [22] based on inconsistency of limb positioning and location of region of interest we used the method introduced by Beck et al when evaluating FN width [23]. This method excludes biologically unlikely values, 3 standard deviations (SD) above or below the mean. This resulted in exclusion of 9 scans of the FN for boys (3 in the car/bus group and 6 in the walking/cycling group) and 12 scans in the FN for girls (2 in the car/bus group and 10 in the walking/cycling group).

The study was conducted according to the Helsinki declaration of 2000 and was approved by the Ethics Committee of Lund University (LU 453-98; 1998-09-15). Informed written consent was obtained from parents or guardians of all participating children prior to study start.

## Results

There were in girls no group differences at baseline when comparing those who walked or cycled to school with those who went by car or bus in life style factors (Table 1), age, anthropometry or BMC (Table 2) while those who walked or cycled had a wider femoral neck (Table 2). Boys who walked and cycled to school had a higher total body lean mass, BMC and L3 width at baseline compared to those who went by car or bus (Table 3).

**Table 1:** Lifestyle factors in children who walked and cycled or travelled by car or bus to school for two years, data are presented as numbers of children with the proportion within each group (in brackets) expressed as % or as mean (SD).

	Girls (n = 65)			Boys (n = 81)		
	
	Walking or cycling	Car or bus	*p*-value	Walking or cycling	Car or bus	*p*-value
**At baseline**						
Numbers	48	17		57	24	
Distance to school (km)	0.5 (0.5)	2.4 (2.5)	<0.001	0.6 (0.6)	2.0(1.5)	<0.001
Excluding diary products	0 (0%)	0 (0%)	1.00	0 (0%)	0 (0%)	1.00
Drinking coffee	1 (2%)	0 (0%)	1.00	1 (2%)	1 (4%)	0.51
Tried to lose weight	1 (2%)	0 (0%)	1.00	0 (0%)	0 (0%)	1.00
Chronic disease	2 (4%)	1 (6%)	1.00	3 (5%)	2 (8%)	0.63
Medication	2 (4%)	2 (12%)	0.28	4 (7%)	1 (4%)	1.00
Fractures	10 (20%)	2 (12%)	0.49	7 (12%)	2 (8%)	0.72
Menarche	0 (0%)	0 (0%)	1.00	---	---	---
Total organized physical activity (hours per week)	0.9 (1.1)	0.8 (0.8)	0.78	1.5 (1.6)	1.4 (1.2)	0.90
						
**During study period**						
Total organized physical activity (hours per week)	3.4 (1.5)	3.5 (1.1)	0.84	4.5 (1.7)	4.1 (2.6)	0.41
						
**At follow-up - Accelerometer data**						
Numbers	46	15		52	20	
Recording time per day (hrs/day)	11.6 (2.0)	12.1 (1.3)	0.34	12.0 (1.3)	12.0 (1.4)	0.95
Mean activity (mean counts/min)	615 (144)	615 (198)	0.99	744 (232)	756 (318)	0.87
Moderate to vigorous activity (min/day)	190 (34)	194 (50)	0.77	210 (50)	206 (49)	0.74
Vigorous activity (min/day)	34 (13)	35 (16)	0.82	46 (25)	46 (19)	0.99

**Table 2:** Baseline data and annual changes evaluating the effect of school transportation mode for girls, anthropometrics, bone mineral content (BMC) and bone width are included, data are presented as mean (SD).

	Girls (N = 65)								
	
	Base line			Annual changes (unadjusted)			Annual changes (adjusted)		
	Walking or cycling	Car or bus	*p*- value	Walking or cycling	Car or bus	*p*- value	Walking or cycling	Car or bus	*p*- value
Numbers	N = 48	N = 17		N = 48	N = 17		N = 48	N = 17	
Age (yrs)	7.9 (0.6)	7.5 (0.5)	0.06	---	---	---			
Weight (kg)	27.8 (5.9)	26.0 (5.0)	0.28	3.6 (1.6)	3.1 (1.3)	0.28			
Height (cm)	128.5 (7.4)	126.6 (4.5)	0.31	5.7 (0.9)	5.7 (0.6)	0.88			
Lean mass (kg)	20.1 (2.9)	19.3 (2.0)	0.25	2.0 (0.6)	1.7 (0.5)	0.19	1.9 (0.4)	1.8 (0.4)	0.14
Fat mass (kg)	5.6 (3.9)	4.6 (3.3)	0.35	1.5 (1.1)	1.4 (1.1)	0.87	1.5 (1.1)	1.4 (1.1)	0.63
**BMC (g)**									
L2-L4	15.4 (3.1)	14.5 (3.0)	0.31	2.0 (0.7)	2.0 (0.8)	0.91	2.0 (0.6)	2.0 (0.6)	0.85
Third lumbar vertebra	5.1 (1.1)	4.8 (1.1)	0.30	0.66 (0.4)	0.74 (0.3)	0.40	0.66 (0.2)	0.72 (0.2)	0.36
Femoral neck	2.7 (0.7)	2.5 (0.4)	0.26	0.29 (0.4)	0.32 (0.2)	0.76	0.29 (0.3)	0.30 (0.3)	0.96
Ward	1.2 (0.6)	1.0 (0.2)	0.18	0.13 (0.3)	0.17 (0.1)	0.67	0.13 (0.3)	0.15 (0.3)	0.89
Trochanter	2.7 (1.5)	2.4 (0.8)	0.47	0.58 (0.6)	0.56 (0.3)	0.91	0.59 (0.6)	0.54 (0.6)	0.76
**Bone Width (cm)**									
Third lumbar vertebra	2.90 (0.28)	2.82 (0.20)	0.28	0.10 (0.07)	0.13 (0.05)	0.22	0.11 (0.06)	0.12 (0.06)	0.33
Femoral neck	2.48 (0.19)	2.36 (0.11)	0.03	0.08 (0.11)	0.06 (0.08)	0.48	0.09 (0.09)	0.03 (0.09)	0.18

**Table 3:** Baseline data and annual changes evaluating the effect of school transportation mode for boys, anthropometrics, bone mineral content (BMC) and bone width are included, data are presented as mean (SD).

	Boys (N = 81)								
	
	Base line			Annual changes (unadjusted)			Annual changes (adjusted)		
	Walking or cycling	Car or bus	*p*- value	Walking or cycling	Car or bus	*p*- value	Walking or cycling	Car or bus	*p*- value
Numbers	N = 57	N = 24		N = 57	N = 24		N = 57	N = 24	
Age (yrs)	8.0 (0.5)	7.8 (0.7)	0.21	---	---	---			
Weight (kg)	28.8 (5.4)	27.0 (4.8)	0.15	3.6 (1.2)	2.8 (1.2)	0.01			
Height (cm)	131.1 (7.0)	128.3 (7.3)	0.10	5.8 (0.7)	5.4 (0.7)	0.02			
Lean mass (kg)	22.5 (3.0)	20.9 (2.8)	0.03	2.3 (0.4)	2.0 (0.4)	<0.01	2.3 (0.4)	2.1 (0.4)	0.10
Fat mass (kg)	4.0 (3.0)	3.7 (2.8)	0.61	1.3 (1.1)	0.8 (0.9)	0.07	1.3 (1.0)	0.9 (1.0)	0.22
**BMC (g)**									
L2-L4	16.5 (3.0)	14.7 (3.6)	0.02	2.1 (0.8)	1.8 (0.6)	0.12	2.1 (0.6)	1.9 (0.6)	0.18
Third lumbar vertebra	5.7 (1.0)	4.9 (1.1)	<0.01	0.59 (0.4)	0.67 (0.3)	0.40	0.65 (0.3)	0.65 (0.3)	0.97
Femoral neck	3.0 (0.7)	2.6 (0.6)	0.01	0.34 (0.3)	0.42 (0.3)	0.29	0.35 (0.3)	0.40 (0.3)	0.41
Ward	1.4 (0.6)	1.1 (0.3)	0.06	0.19 (0.2)	0.24 (0.3)	0.36	0.18 (0.2)	0.26 (0.3)	0.19
Trochanter	2.8 (1.5)	2.7 (1.2)	0.63	0.47 (0.4)	0.45 (0.3)	0.80	0.45 (0.4)	0.48 (0.4)	0.78
**Bone Width (cm)**									
Third lumbar vertebra	3.10 (0.23)	2.99 (0.27)	0.05	0.08 (0.09)	0.10 (0.07)	0.42	0.10 (0.07)	0.09 (0.07)	0.40
Femoral neck	2.51 (0.20)	2.43 (0.10)	0.62	0.09 (0.08)	0.10 (0.06)	0.62	0.09 (0.07)	0.11 (0.07)	0.19

The annual increases in BMC or bone width was no different in neither girls nor boys when comparing those who walked or cycled to school with those who went by car or bus (Table 2 and Table 3). The same outcome was found both before and after adjustment for possible confounders (Table 2 and Table 3).

Neither the objective nor the subjective duration of physical activity differed when comparing girls and boys who walked or cycled to school with those who went by car or bus (Table 1). All children, independent of having an active or passive school transportation, reached above the by the United Kingdome Expert Consensus Group recommended level of 60 minutes of moderate to intense physical activity per day [21].

## Discussion

An active mode of transportation to school for two years did in this cohort of pre- pubertal boys and girls not provide any additional benefits in skeletal development in comparison with a passive mode of school transportation. However, it is most essential to emphasize that this study focuses on skeletal development and does not evaluate other possible health benefits reached by an active transportation to school.

There are several conditions that could explain the fact that active school transportation was not associated with additional benefits in the accrual of BMC or gain in bone width. As supported by the accelerometer data, all the children in this study reached above the internationally recommended level of 60 minutes of moderate to vigorous activity per day [21] (Table 1). It is also well described that a more intense activity than walking or cycling is more important for the skeletal development than a long duration of repeated low intense physical activity [24-26]. As to produce the most pronounced osteogenic stimuli, a mechanical load should be high in magnitude, fast with unusually distributed strains [27-30]. That is, high intensity activities like tennis, soccer and weight-lifting provides pronounced improvements in BMC [31-33] whereas endurance activities like cycling, long distance running and swimming provides no or only minor skeletal benefits [25,34-36].

Furthermore, the mean distances to school in those who walked or cycled were only in mean 500 metres in girls and 600 metres in boys. That is, the contribution of active school transportation for the total daily amount of physical activity is low. This fact is supported by some data in the literature, inferring that the mode of transportation to school in 5 year old children in the UK does not affect the overall physical activity [37]. It must also be realised that compared to the effect of growth and genetic factors, the mechanical load provided by habitual daily physical activity only provides minor additional skeletal benefits [38]. Also the timing of the mechanical stimuli could affect our results. It is generally accepted that the skeleton is most susceptible to mechanical stimuli in the late pre- or early peri- pubertal period, corresponding to Tanner stages 2 and 3 [32,39], while the children in this study remained in Tanner stage 1 during the entire period.

### Study Strengths

The population-based study design increase the ability to draw generalized inferences as do the fact that there were no differences in height, weight or body mass index (BMI) when comparing the study participants and the non-participants in the population based invited children [10,11]. The use of accelerometers to objectively evaluate levels of physical activity instead of just relying on subjective estimations by questionnaires is also an advantage [40,41].

### Study limitations

It had been advantageous if we had been able to provide data separately for those who walked, cycled, went by car and went by bus but this was not possible due to the risk of making type II errors. The classification of the mode of school transportation was entirely based on the questionnaires without any objective registration of school transportation. This was not a randomized controlled trial. Instead the mode of transportation was self-selected. But, as the choice of school transportation predominantly was based on distance to school than the phenotype of the child, this decreases the risk of selection bias. The group differences in BMC and bone size that existed in some regions at baseline are potential confounding factors. However, as the results remained after adjustment for these possible confounders, it seems less probable that the confounders influenced our inferences. The fact that around half of the children had extra physical education classes in school could also influence the results. However, as there were no differences between the groups regarding the total amount of organized physical activity (organized physical training at school and during spare time) or in accelerometer data, this indicate the problem to be of no biological importance.

## Conclusion

The mode of transportation to schools for two years seems not to influence the accrual of bone mineral or bone width in these 7-9 years old Swedish children, possibly as the level of everyday physical activity in this study-population is so high that walking and cycling to school contributes little to the total amount of activity. If active school transportation provide skeletal benefits in cohorts with a general lower level of activity and a longer distance to school, ought to be evaluated in future trials.

## Competing interests

The authors declare that they have no competing interests.

## Authors' contributions

BL carried out the statistical analysis, the interpretation of data and the writing of the manuscript. SSL collected all the data except the accelerometer data and were involved in the drafting of the manuscript. MD collected and calculated the accelerometer data and was involved in the drafting of the manuscript. MK designed the study, worked with the analysis and the interpretation of data and was in charge of writing the manuscript. All authors contributed intellectually to the manuscript and all authors have read the manuscript and approved the submission.

## Pre-publication history

The pre-publication history for this paper can be accessed here:

http://www.biomedcentral.com/1471-2474/11/25/prepub
